# The pre‐analytical stability of 25‐hydroxyvitamin D: Storage and mixing effects

**DOI:** 10.1002/jcla.23037

**Published:** 2019-10-06

**Authors:** Anwar Borai, Haitham Khalil, Basma Alghamdi, Raghad Alhamdi, Najwa Ali, Suhad Bahijri, Gordon Ferns

**Affiliations:** ^1^ King Abdullah International Medical Research Center King Saud bin Abdulaziz University for Health Sciences Pathology King Abdulaziz Medical City Jeddah Saudi Arabia; ^2^ Department of Clinical Biochemistry Faculty of Medicine King Abdulaziz University Jeddah Saudi Arabia; ^3^ Division of Medical Education Brighton and Sussex Medical School Mayfield House Brighton UK

**Keywords:** effect, mixing, pre‐analytical, stability, storage, vitamin D

## Abstract

**Background:**

There is an increasing demand for serum 25‐OH VitD testing globally, and this has led to the greater use of automated immunoassays. These may be more prone to non‐specific interference, that is thought to be related to pre‐analytical stability of biological samples. We have investigated the changes in serum 25‐OH VitD concentrations that are caused by storage and mixing conditions, and if such changes are statistical, or clinically important.

**Methods:**

Blood samples were collected into plain tubes from 31 healthy donors. After separation, serum samples were stored at −20°C and analysis was carried out with and without mixing (vortexing) at different time intervals of days (0, 1, 2, 3, 4, 5, 15, and 30). All samples were analyzed using a chemiluminescent immunoassay.

**Results:**

Mean serum 25‐OH VitD concentrations for subsequent days of storage compared with day 0 showed a significant time effect (*P* < .05) except for the samples on day 1 (*P* = .654) in non‐vortexed samples and day 2 (*P* = .087), 5 (*P* = .118) and 30 (*P* = .118) in vortexed samples. Comparing values for vortexed and non‐vortexed samples on the same day, serum 25‐OH VitD showed a significant difference on days 1 (*P* = .003), 4 (*P* = .037), 5 (*P* = .002), and 30 (*P* = .025). However, the maximum change value was 8.85% which was less than the known total allowable error (TEa) and reference change value (RCV) for serum 25‐OH VitD.

**Conclusion:**

25‐OH VitD is pre‐analytically stable after long‐term sample storage at −20°C and can be analyzed without vortexing. This may be beneficial for both research and diagnostic laboratories.

## 1 INTRODUCTION

Vitamin D is required for normal development and bone health. Vitamin D deficiency is known to cause rickets in children and osteomalacia in adults. Furthermore, recent studies indicate that vitamin D status is implicated in the development of diabetes mellitus^1^ and other chronic conditions.^2^ Vitamin D deficiency has been reported to be common in many countries.^3^, ^4^ These findings have led to an increase in vitamin testing worldwide, which has placed considerable demands on clinical laboratories.

It is possible that pre‐analytical factors may affect the reliability of serum 25‐hydroxy vitamin D (25‐OH VitD) estimations, and this is important if appropriate clinical interventions are to be made. Hence, it is important to know how pre‐analytical factors may affect the results of testing. These factors include sample stability on storage and processing of samples after defrosting. This would be particularly important when analysis is not carried out on a daily basis, and samples are frozen for various lengths of time before estimation. However, to date, there are limited published data on pre‐analytical stability of 25‐OH VitD in human blood following storage at various temperatures, and for various periods. One study focused on the effects of repeated freeze‐thaw cycles.^5^ Another study investigated the stability of serum 25‐OH VitD levels over time under various sample storage conditions using specimens from only eight patients**.** 6 A recent study examined the effect of storage at −20°C in the primary collection tube on 25‐OH VitD measured by immunoassay.^7^ These three studies and other studies (Table 1) have reported vitamin D to be a stable analyte on storage with no concern about using the quality requirement parameters to estimate the clinical decision thresholds. However, these studies have been limited either by the small numbers of specimens examined or by the chosen time intervals for storage. Furthermore, no previous study has examined the effects of whether samples were mixed following thawing on 25‐OH VitD stability. Vortex mixing is usually used to ensure homogenity of sample following thawing of frozen samples, adding an additional step to the analytical process.

**Table 1:** Summary of previous studies about 25‐OH VitD stability

Publication	No. of samples	Storage Temp.	Analyzer	Type of Investigation	Tube type	Period	Outcome
Wielders et al, 2009^6^	8	‐20°C, Dark 6°C Dark RT Bench RT	Cobas E601 (Roche)	Temperature and freeze‐thawing cycles	Plain (serum)	Up to 2 mo	Frozen and unfrozen serum samples are reliable for analysis up to 3 d. No effect of light exposure or freeze‐thawing cycles on 25‐OH VitD levels.
Antoniucci, D. M et al, 2005.8^8^	20	‐70°C RT	RIA (DiaSorin)	Freeze‐thawing cycles	Plain (serum)	3 d	After thawing and refreezing up to four times 25‐OH VitD results are still reliable.
Hayden Y et al, 2015^7^	19	‐20°C	Cobas E602	Storage, temperature, and sample type	SST (serum)	144 d	25‐OH VitD can be stored in the primary SST at –20°C without statistically significant change.
Bozkurt et., 2018^13^	153	RT 2‐8°C ‐20°C ‐40°C	IDS‐ISYS	Temperature and storage time	SST (serum)	3 mo	There was no significant statistical difference in 25‐OH VitD level when they compared with first day sample level.
Chu‐Ling Yu 2010^14^	20	RT, 2‐8°C	Liaison (DiaSorin) and RIA	Delay in separation, sample type, and storage time	Plain (serum), EDTA (plasma), Heparin (plasma)	NA	No need for immediate analysis of blood samples after collection or for the choice of a tube type. The 25‐OH VitD in heparinized plasma may be higher than in serum or EDTA plasma
Ayfer Colak et al, 2013^15^	15	RT 2‐8°C ‐20°C ‐80°C	Cobas E 411	Storage conditions and sample type	SST (serum) and EDTA (plasma)	3 mo	There is no statistically significant difference between serum and plasma 25‐OH VitD at different storage conditions.
Borai A., 2016^8^	50	−80°C	HPLC, Architect (Abbott), and Liaison (Diasorin)	Sample type	SST (serum) and Plain (serum)	6 mo	The gel in SST does not interfere with the measurement of 25‐OH Vit D.

Note

Immunoassay principles: Cobas, electrochemiluminescence; RIA, radioimmunoassay; IDS‐ISYS; chemiluminescent; Architect, chemiluminescent; Liaison, chemiluminescent.

Abbreviations: RT, room temperature; SST, serum separator tube, HPLC, high pressure liquid chromatography.

Therefore, the aim of the present study was to assess the stability of 25‐OH VitD over a longer period of time and to investigate the effect of vortex mixing on the results of serum 25‐OH VitD analysis. In addition to this, our study aimed to investigate whether the potential changes in the results are clinically significant or not, as this has not been discussed in the previous studies.

## 2 METHODOLOGY

### 2.1 Subjects identification

Subjects were recruited randomly for the study from healthy blood donors at King Abdulaziz Medical City, Western Region (National Guard Health Affairs (NGHA)) without regards to vitamin D intake. The study was approved by the Institutional Review Board (IRB) at King Abdullah International Medical Research Center (KAIMRC) for ethical considerations (SP18/427/J). All participants signed an informed consent form after explaining the purpose of the study, and being assured that it imposed no additional risk. Blood samples were drawn in the phlebotomy area of the laboratory department, King Saud Bin Abdulaziz University for Health Sciences (KSAU‐HS).

### 2.2 Specimens collection

All specimens were obtained on a single day. Blood was collected from each subject into two plain (red top) tubes (Becton‐Dickinson) using standard venesection technique. Samples with insufficient volume (<8 mL of blood), lipemia, or hemolysis were excluded from the study. Specimens were centrifuged within 1 hour of collection, and the serum separated and aliquoted into 1.0 mL Eppendorf tube with 500 µL of serum. Aliquots were stored at −20°C except for two per subject that were analyzed on the day of collection (day 0). One aliquot was vortexed for 10 seconds, and the second analyzed without vortexing. This procedure was repeated using the stored aliquots on days 1, 2, 3, 4, 5, and 30. All specimens were measured for total 25‐OH VitD concentration using a chemiluminescent immunoassay (CLIA) on an Architect i2000 analyzer. The method is a direct competitive assay (Ag‐Ab reaction), which detects both active forms of vitamin D (25‐OH VitD2 and 25‐OH VitD3).

The assay is standardized immunoassay and traceable to national institute of standards and technology (NIST). The coefficient of variation (CV) values for intra‐assay (1.4%‐3.7%) and inter‐assay (2.7%‐4.6%) were published in our previous study.^8^ The laboratory at King Abdulaziz Medical City is accredited by the College of American Pathologists. It participates in a rigorous program of internal quality control and external quality assessment. All internal quality control results and calibration were within the acceptable limits in each individual day of research samples analysis.

### 2.3 Statistical analysis

Statistical analysis was done using SPSS software version 22. Comparison between group means was performed using paired *t* tests and across different groups using analysis of variance (ANOVA). A two‐tailed *t* test was conducted using an *α* level of .05 for all statistical tests.

## 3 RESULTS

The study comprised 31 subjects, with a mean age ± SD of 35 ± 15 years and 20 of whom were males (64.5%). Mean weight and height ± SD reported were 65 ± 16 kg and 1.62 ± 0.09 m, respectively. Four subjects were taking vitamin D supplements. The mean serum 25‐OH VitD estimated values on different days, with and without vortexing are shown in Table 2. The minimum and maximum 25‐OH VitD levels of the baseline non‐vortexed and vortexed samples were 15.7 and 67.4 nmol/L, respectively.

**Table 2:** Mean concentration of 25‐OH VitD in analyzed samples at different time intervals

Day	Non‐vortexed				Vortexed				*P* *
	Mean ± SD (nmol/L)	Subsequent days to day 0			Mean ± SD (nmol/L)	Subsequent days to day 0			
		*Diff.*		*P*		*Diff.*		*P*	
		Bias	%			Bias	%		
0	34.12 ± 12.97				33.66 ± 12.97				.118
1	34.26 ± 13.72	0.14	0.41	.654	35.10 ± 13.19	0.75	4.28	.043	.003
2	37.02 ± 13.20	2.90	8.50	.000	35.85 ± 13.01	1.50	6.51	.087	.171
3	35.18 ± 13.29	1.06	3.11	.039	35.47 ± 13.04	1.12	5.38	.039	.473
4	35.73 ± 12.89	1.61	4.72	.000	36.65 ± 13.35	2.30	8.88	.000	.037
5	36.54 ± 13.97	2.42	7.09	.000	34.98 ± 13.17	0.63	3.92	.118	.002
15	36.99 ± 13.22	2.87	8.41	.000	36.55 ± 13.75	2.20	8.59	.004	.499
30	35.99 ± 12.63	1.87	5.48	.000	34.05 ± 12.71	0.58	1.16	.118	.025

Note

Diff. refers to the mean difference in serum 25‐OH VitD results on subsequent days compared with day 0 with the *P* value of the corresponding day.

* *P* refers to the difference in results between non‐vortexed and vortexed specimens.

### 3.1 Storage effect

**Figure 1:**
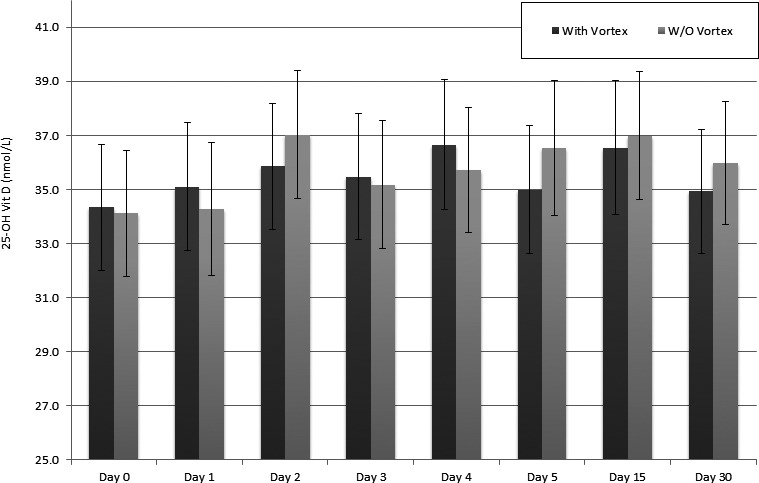
Visual summary of changes in mean concentrations ± SE of 25‐OH VitD with and without vortex in each day of analysis

For non‐vortex mixed samples, the mean concentration of 25‐OH VitD on day 0 was not found to differ significantly on day 1, (*P* = .654). However, on day 2, the level was significantly higher (*P* < .001) by 8.50%. This was followed by fluctuations in the mean value on subsequent days, with a slight decrease in the mean on day 3, then a slight increase on day 5, but in all cases, the mean remained significantly higher compared with the mean at day 0 (Table 2). An ANOVA for the results of the non–vortex‐mixed specimens indicated a significant time effect, *Wilks’ Lambda* = 0.225, *F* = 11.83, *P* < .05, *η* ^2^ *= *0.77. This indicated that there was a significant trend toward a rise in mean concentration of 25‐OH VitD compared with day 0 (fresh specimens) over time (Figure 1).

Similarly, there was a clear difference in the measured concentrations of mean serum 25‐OH VitD throughout all storage days for the vortex‐mixed serum specimens compared with day 0 mean (Figure 1). There were also significant differences (*P* < .05) in mean values on day 1, 3, 4, and 15 compared with day 0 (Table 2). An ANOVA of vortex‐mixed specimens showed a significant difference in the mean values measured at different times, *Wilks’ Lambda* = 0.48, *F* = 3.76, *P* < .05, *η* ^2^ *= *0.52. Although this indicates that there was a significant trend toward a rise in mean concentration of serum 25‐OH VitD compared with day 0 (fresh specimens) over time but this trend was not as apparent as found in the non‐vortexed samples (Figure 1).

### 3.2 Mixing (vortexing) effect

Comparing mean levels between non‐vortexed and vortexed specimens that were measured on the same day, there were significant differences (*P* < .05) between them on days 1, 4, 5, and 30, but not on days 0, 2, 3, and 15 (*P* > .05; Table 2).

## 4 DISCUSSION

The literature provides useful information regarding 25‐OH VitD pre‐analytical stability using different analytical methods and experimenting with different storage conditions.^5^, ^6^, ^7^ Previous studies suggest that serum 25‐OH VitD is a stable analyte under various conditions, with no significant change in 25‐OH VitD concentration was observed under multiple freeze‐thaw cycles, light exposure, or different storage conditions.

On the other hand, having the advantage of a larger population sample size, our study showed that there was a statistically significant difference in serum 25‐OH VitD levels when measured with or without sample vortexing over different time intervals, up to 30 days in samples stored at −20°C. We assume that these statistical differences may be associated with sample homogeneity in the vortexed serum samples compared with the non‐vortexed samples and sample evaporation for the day‐by‐day analysis due to storage.

The reason for the statistical significant differences in our outcomes compared with previous studies 5, 6, 7 is unclear. However, they may result from the larger population sample of our study, and the differences in their local protocols, that is, short time intervals while our protocol has been extended up to 30 days of storage.

Data on the biological variability of analyte concentrations have important implications in clinical chemistry, including assessment of the significance of differences between consecutive results obtained in a single patient.^9^ The most widely accepted approach for this purpose is use of the so‐called reference change value (RCV) described by Harris and Brown in 1979.^10^

Brescia's study suggests that individual biological variability and analytical variability may affect the variability of 25‐OH VitD analysis.^11^ They used the DiaSorin LIAISON chemiluminescence immunoassay, which is the same principle to our assay using Architect i2000, to assess single individual, intra‐individual biological variability, and analytical variability of 25‐OH VitD.^11^ They assessed the variation in serial measurements of 25‐OH VitD samples, and they concluded that the reference change value (RCV) was 18.0%.^11^

Thus, differences in values not exceeding the RCV have no clinical significance on patient outcomes. Our results showed that differences on each subsequent days, for non‐vortexed and vortexed specimens, did not exceed the maximum value of 8.88%. While most of the differences obtained in our study were statistically significant, but at the same time, they were not considered as clinically significant as it did not exceed the recommended RCV for 25‐OH VitD of 18%.^11^

The allowable total error (TEa) is another quality parameter. It refers to the amount of error that is allowable without invalidating the interpretation of a test result or reaching the clinical decision thresholds. It can be expressed as the maximum allowable bias and imprecision which can be calculated for each analyte. The TEa for 25‐OH Vit D is 46%. It means that the value with a bias of more than 46% from the original value will be significant. This value was calculated by the Advisory Panel of Vitamin D External Quality Assessment Scheme (DEQAS),^12^ and it was based on the most common proficiency testing programs. In our study, the maximum difference between intervals, mixing, and without mixing measurements was 8.88%. This value was less than the TEa for 25‐OH Vit D.

Therefore, our data show that it is unnecessary to vortex specimens before the measurement of 25‐OH VitD. This is a useful practical observation because time to finish the batches of high number of samples in the clinical laboratory can be decreased by omitting the vortex step. Productivity in the clinical laboratory will increase as well. The data showed that, when measuring serum 25‐OH VitD, carrying out analysis can be on the day of collection or the following days as changes in 25‐OH VitD did not exceed the recommended mentioned RCV and TEa. Even with normal storage condition of −20°C, serum 25‐OH VitD concentration is stable in all samples analyzed during the following days. This feature of stability is important for the purpose of 25‐OH VitD batching analysis in both research studies and diagnostic settings.

In addition to this, our outcomes show that there is no need for <−20°C storage condition (eg, <−75°C freezer) to keep intact samples integrity for testing 25‐OH VitD for up to 1 month period.

This study had the limitation that the RCV was obtained using the LIAISON, Diasorin analyzer, alone. Therefore, it is suggested in the future to obtain the RCV on Abbott, Architect i2000 as this was not provided in previous literatures. Another limitation that the study was designed to include healthy subjects only but those with other pathological conditions with extreme 25‐OH VitD values were not considered.

## 5 CONCLUSION

In conclusion, delayed analysis and running samples without vortexing have no “clinically significant” effect on 25‐OH VitD level. Such outcomes can save time and efforts which are important factors in both diagnostic and research laboratories.

## AUTHOR CONTRIBUTION

AB researched literature and conceived the study. Recruitment of subjects and analytical work was carried out by BG, RA, HK, and NA. SB and GF reviewed all drafts of the manuscript. All authors reviewed and edited the manuscript and approved the final version of the manuscript.

## ETHICAL APPROVAL

The study was approved by the Research Ethics Committee at King Abdullah International Medical Research Center, Jeddah, Saudi Arabia.

## GUARANTOR

Dr A Borai guarantor for this paper.
